# Exposure of *Aspergillus fumigatus* to *Klebsiella pneumoniae* Culture Filtrate Inhibits Growth and Stimulates Gliotoxin Production

**DOI:** 10.3390/jof9020222

**Published:** 2023-02-08

**Authors:** Aaron Curtis, Michelle Ryan, Kevin Kavanagh

**Affiliations:** Medical Mycology Unit, Department of Biology, Maynooth University, W23 F2H6 Co. Kildare, Ireland

**Keywords:** *Aspergillus fumigatus*, *Klebsiella pneumoniae*, fungal–bacterial interaction, gliotoxin, proteomics

## Abstract

*Aspergillus fumigatus* is an opportunistic fungal pathogen capable of inducing chronic and acute infection in susceptible patients. *A. fumigatus* interacts with numerous bacteria that compose the microbiota of the lung, including *Pseudomonas aeruginosa* and *Klebsiella pneumoniae*, both of which are common isolates from cystic fibrosis sputum. Exposure of *A. fumigatus* to *K. pneumoniae* culture filtrate reduced fungal growth and increased gliotoxin production. Qualitative proteomic analysis of the *K. pneumoniae* culture filtrate identified proteins associated with metal sequestering, enzymatic degradation and redox activity, which may impact fungal growth and development. Quantitative proteomic analysis of *A. fumigatus* following exposure to *K. pneumoniae* culture filtrate (25% *v*/*v*) for 24 h revealed a reduced abundance of 1,3-beta-glucanosyltransferase (−3.97 fold), methyl sterol monooxygenase erg25B (−2.9 fold) and calcium/calmodulin-dependent protein kinase (−4.2 fold) involved in fungal development, and increased abundance of glutathione S-transferase GliG (+6.17 fold), non-ribosomal peptide synthase GliP (+3.67 fold), O-methyltransferase GliM (+3.5 fold), gamma-glutamyl acyltransferase GliK (+2.89 fold) and thioredoxin reductase GliT (+2.33 fold) involved in gliotoxin production. These results reveal that exposure of *A. fumigatus* to *K. pneumoniae* in vivo could exacerbate infection and negatively impact patient prognosis.

## 1. Introduction

A wide range of microbes can be present in the lung as commensals or pathogens, and there is significant microbial diversity present [1]. There is reduced microbial diversity in the sputum of patients with acute exacerbations of chronic obstructive pulmonary disease, indicating a poor prognosis, and the presence of *Staphylococcus* and absence of *Veillonella* in sputum is associated with a high one-year mortality risk in patients [2]. In addition, coinfection with *Pseudomonas aeruginosa* and *Aspergillus fumigatus* has been implicated in a worsened disease state in Cystic Fibrosis (CF) patients, with each species stimulating the growth and colonisation of the other [3]. The bacterium *Klebsiella pneumoniae* and the fungus *A. fumigatus* are commonly isolated from the lungs of CF patients [4], indicating a similar interaction could occur, impacting disease development in patients.

*K. pneumoniae* is a Gram-negative rod-shaped bacteria that is responsible for approximately one-third of all Gram-negative infections worldwide [5]. Pneumonia caused by *K. pneumoniae* is characterised by a strong inflammatory response which is due to the production of pro-inflammatory cytokines in addition to high neutrophil and macrophage counts [6]. Within the host, *K. pneumoniae* is found in the gastrointestinal tract and the upper respiratory tract, where it is most frequently acquired through nosocomial acquisition [7]. The first report of a CF patient becoming infected with a colistin-resistant strain of *K. pneumoniae* was in 2014 [8], and since then, the number of reports of critically ill patients becoming infected with such strains has increased.

The filamentous fungus *A. fumigatus* is widespread in the environment and can cause three types of infection in susceptible individuals. Allergic bronchopulmonary aspergillosis (ABPA) arises due to an allergic immune response to *A. fumigatus* stimulating T-helper cells to recruit immune cells, particularly eosinophils, resulting in inflammation of pulmonary tissue. Patients with asthma or CF are among the most susceptible to this form of aspergillosis, and early diagnosis of the disease is important for avoiding complications such as pulmonary fibrosis [9]. Chronic pulmonary aspergillosis (CPA) occurs when pre-damaged pulmonary tissue becomes colonised by *A. fumigatus* [10]. The colonisation results in the formation of an aspergilloma, or fungal ball, which can result in severe haemoptysis [11]. Invasive aspergillosis is the most serious form of aspergillosis, with a one-year survival rate for solid organ transplant patients of 59%, while the rate for stem cell transplant recipients can be as low as 25% [12]. Patients suffering from neutropenia were once considered to be the main target group for this type of aspergillosis; however, non-neutropenic patients such as transplant patients, AIDS patients and ICU patients are also susceptible to infection [13].

The interaction between *A. fumigatus* and *P. aeruginosa* has been characterised previously, indicating decreased fungal growth and increased gliotoxin production in the presence of bacterial cells. In contrast, exposure of *A. fumigatus* hyphae to *P. aeruginosa* culture filtrate led to increased growth and decreased gliotoxin production [14], and secreted products of *A. fumigatus* can promote *P. aeruginosa* growth in nutrient-poor conditions [15]. *K. pneumoniae* is often found in polymicrobial interactions with *A. fumigatus* within the lung, and these interactions may be antagonistic or synergistic [16]. For example, *K. pneumoniae* is capable of inhibiting spore germination and hyphal development in different *Aspergillus* spp., and this effect was dependent upon the direct physical interaction between the bacteria and the fungus [17]. *A. fumigatus* and *K. pneumoniae* can co-exist in the lungs of immunocompromised patients, but the interaction between these two pathogens is poorly characterised. The aim of the work presented was to characterise the effect of *K. pneumoniae* culture filtrate on the growth and proteomic response of *A. fumigatus*, as this might give an insight into an important bacterial-fungal interaction in the lungs of infected patients.

## 2. Materials and Methods

### 2.1. A. fumigatus Growth Conditions

*Aspergillus fumigatus* ATCC 26933 was grown on malt extract agar plates at 37 °C for a minimum of 96 h, and conidia were harvested by washing with 0.1% (*v*/*v*) Tween-20 and phosphate buffered saline (PBS-T). Czapek–Dox liquid medium (Duchefa Biochemie) (50 mls) was inoculated with conidia at a density of 1.2 × 10^6^/mL.

### 2.2. Preparation of the Bacterial Culture Filtrate

*K. pneumoniae* ATCC 13439 was grown on nutrient agar plates for 96 h. Czapek–Dox broth was inoculated with bacteria and incubated at 37 °C and 200 rpm for 96 h. Cells were harvested by centrifugation for 15 min at 1258× *g* at room temperature, and the culture filtrate was filter sterilised using 0.45 μm filtropur S filters. Culture filtrates were stored at −20 °C until required.

### 2.3. Assessment of Fungal Biomass

*A. fumigatus* was grown for 48 h in Czapek–Dox broth prior to the addition of *K. pneumoniae* culture filtrate or PBS (n = 3) at a concentration of 25% *v*/*v*. The cultures were incubated at 37 °C and 200 rpm. The mycelia were harvested after 24 h using Miracloth (Millipore). The wet weight of the mycelia was then measured using a weighing scale and expressed as weight in grams.

### 2.4. Gliotoxin Extraction and Quantification by RP-HPLC

*K. pneumoniae* culture filtrate was added to the 48 h old *A. fumigatus* cultures at a final concentration of 25% *v*/*v* and incubated at 37 °C and 200 rpm for a further 24 h. Culture filtrate (20 mL) was mixed continuously with an equal volume of chloroform for 2 h. The chloroform layer was removed and evaporated to dryness, and the extract was dissolved in 500 μL HPLC grade methanol. Gliotoxin was quantified using Reverse-Phase HPLC. The mobile phase was 34.9% (*v*/*v*) acetonitrile, 0.1% (*v*/*v*) trifluoroacetic acid (TFA) and 65% (*v*/*v*) HPLC-grade deionised water. Gliotoxin extract (20 μL) was injected into an Agilent ZORBAX SB-Aq 5 μm polar LC column. A standard curve of peak area vs. gliotoxin concentration was generated using gliotoxin standards (Merck) dissolved in HPLC-grade methanol.

### 2.5. Extraction of Protein from K. pneumoniae Culture Filtrate

*K. pneumoniae* was grown for 96 h in Czapex Dox broth. Cells were harvested by centrifugation for 15 min at 1258× *g* at room temperature. The culture filtrate was passed through a 0.45 mm filtropur S filter. The culture filtrate was centrifuged at 14,500× *g* for 10 min to remove any remaining debris, and protein was acetone precipitated overnight at a ratio of 1:5 culture filtrate to acetone. Samples were processed in the same manner as the fungal mycelial samples for proteomic analysis.

### 2.6. Protein Extraction from A. fumigatus Exposed to Bacterial Culture Filtrate

*A. fumigatus* cultures (48 h growth) were supplemented with *K. pneumoniae* culture filtrate (25% *v*/*v*) for 24 h at 37 °C in Czapek–Dox media (n = 3 per group). Hyphae were harvested by filtration, snap-frozen in liquid nitrogen and ground to a fine dust in a mortar using a pestle. Lysis buffer [8 M urea, 2 M thiourea, and 0.1 M Tris-HCl (pH 8.0) dissolved in HPLC-grade dH2O], supplemented with protease inhibitors [aprotinin, leupeptin, pepstatin A, Tosyllysine Chloromethyl Ketone hydrochloride (TLCK) (10 µg/mL) and phenylmethylsulfonyl fluoride (PMSF) (1 mM/mL)] was added (4 mL/g of hyphae). The lysates were sonicated (Bandelin Senopuls) three times for 10 s at 50% power. The cell lysate was subjected to centrifugation (Eppendorf Centrifuge 5418) for 8 min at 14,500× *g* to cellular pellet debris. The protein concentration was quantified by the Bradford method, and samples (100 µg) were subjected to overnight acetone precipitation.

### 2.7. Label-Free Mass Spectrometry (LF/MS)

Following centrifugation for 10 min at 14,500× *g*, acetone was removed, and the protein pellet was resuspended in 25 µL sample resuspension buffer (8 M urea, 2 M thiourea, 0.1 M tris-HCL (pH 8.0) dissolved in HPLC grade dH2O). An aliquot of 5 µL was removed from each of the samples and quantified by the Qubit quantification system (Invitrogen). Ammonium bicarbonate (125 µL, 50 mM) was added to the remainder of the samples. Reduction of the sample was initiated by adding 1 µL 0.5 M dithiothreitol (DTT). The protein samples were then incubated for 20 min at 56 °C before alkylation with 2.7 µL 0.55 M iodoacetamide; this occurred at room temperature in the dark for 15 min. Protease max surfactant trypsin enhancer (Promega) (1 µL, 1% *w*/*v*) and sequence grade trypsin (ThermoFisher Scientific, Cork, Ireland) (0.5 µg/µL), were added to the proteins, respectively, and incubated for 18 h at 37 °C. TFA (1 µL, 100%) was added to each sample to end digestion. The samples were incubated for 5 min at room temperature and centrifuged for 10 min at 14,500× *g*. Samples were purified for mass spectrometry using C18 spin columns as per the manufacturer’s instructions. A speedy vac concentrator was used to dry the peptides, and samples were resuspended in 2% *v*/*v* acetonitrile and 0.05% *v*/*v* TFA and sonicated for 5 min to help with this resuspension. The resulting final peptide concentration was (750 ng/2 µL).

### 2.8. Mass Spectrometry and the Parameters for A. fumigatus and K. pneumoniae Culture Filtrate Proteomic Data Procurement

The digested *K. pneumoniae* culture filtrate sample (500 ng) or *A. fumigatus* protein samples (750 ng) were each loaded onto a QExactive (ThermoFisher Scientific) high-resolution mass spectrometer which was connected to a Dionex Ultimate 3000 (RSlCnano) chromatography system. An increasing acetonitrile gradient was used to separate the peptides in the samples. This gradient was created on a 50 cm EASY-Spray PepMap C18 column with a 75 mm diameter using a 133 min reverse phase gradient at a flow rate of 300 nL/min. All of the data were obtained while the mass spectrometer was functioning in an automatic dependent switching mode. A quantitative analysis of the *A. fumigatus* proteome after exposure to bacterial culture filtrate was conducted using MaxQuant version 1.5.3.3 (http://www.maxquant.org accessed on 14 September 2022) using the settings outlined previously [14]. The search algorithm Andromeda included in the MaxQuant software was incorporated in the correspondence between MS/MS data and the Uniprot-SWISS-PROT database *Neosartorya fumigata* reference proteome obtained from a UniProt-SWISS-PROT database to identify proteins (9647 entries, downloaded July 2022). *K. pneumoniae* culture filtrate was analysed through proteome discoverer v 2.5, and proteins were searched against the UniProtKB database (*Klebsiella Pneumoniae* strain 342, 5738 entries, downloaded September 2022).

### 2.9. Data Analysis of A. fumigatus and K. pneumoniae Culture Filtrate Proteomes

Qualitative analysis of the proteome of the *K. pneumoniae* culture filtrate was investigated using Proteome Discoverer 2.5 and Sequest HT (SEQUEST HT algorithm; Thermo Scientific). Identified proteins were searched against the UniProtKB database (*Klebsiella Pneumoniae* strain 342, 5738 entries, accessed 16 September 2022). Search parameters applied for protein identification were as follows: (i) enzyme name—trypsin, (ii) an allowance of up to two missed cleavages, (iii) peptide mass tolerance set to 10 ppm, (iv) MS/MS mass tolerance set to 0.02 Da, (v) carbamidomethylation set as a fixed modification and (vi) methionine oxidation set as a variable modification. Peptide probability was set to high confidence (with an FDR ≤ 0.01% as determined by Percolator validation in Proteome Discoverer). Peptides identified by 2 or more unique peptides were retained for analysis.

Perseus v.1.6.15.0 was employed to analyse *A. fumigatus* proteomic data, as well as to process and visualise the data. Measurement of protein abundance was based on normalised LFQ intensity values. The data was initially filtered for the removal of contaminants before LFQ intensity values were Log2 transformed, and each sample was placed in its relative group. Proteins which were not found in three out of three replicates in at least one group were excluded from further analysis. Normal intensity values were also used for principle component analysis (PCA). Proteins which were distinctively expressed in one group compared to another or those which were completely absent in one group were noted and included for all further analysis. Gene Ontology (GO) mapping was also conducted using the UniProt gene ID to gain more knowledge about the biological processes and molecular processes of the identified proteins. To gain a better visualisation of the variances between the samples, pairwise student T-tests were conducted for all data using a cut-off of *p* < 0.05. The generation of a volcano plot was done by plotting the log2 fold change values on the x-axis and the log *p*-values on the y-axis, which allowed for pairwise comparison. The top 20 most increased and decreased in abundance proteins identified in the groups were included in these volcano plots. The mass spectrometry proteomics data have been deposited to the ProteomeXchange Consortium via the PRIDE [18] partner repository with the dataset identifier PXD037833.

## 3. Results

### 3.1. Analysis of the Effects of K. pneumoniae Culture Filtrate on A. fumigatus

**Figure 1:**
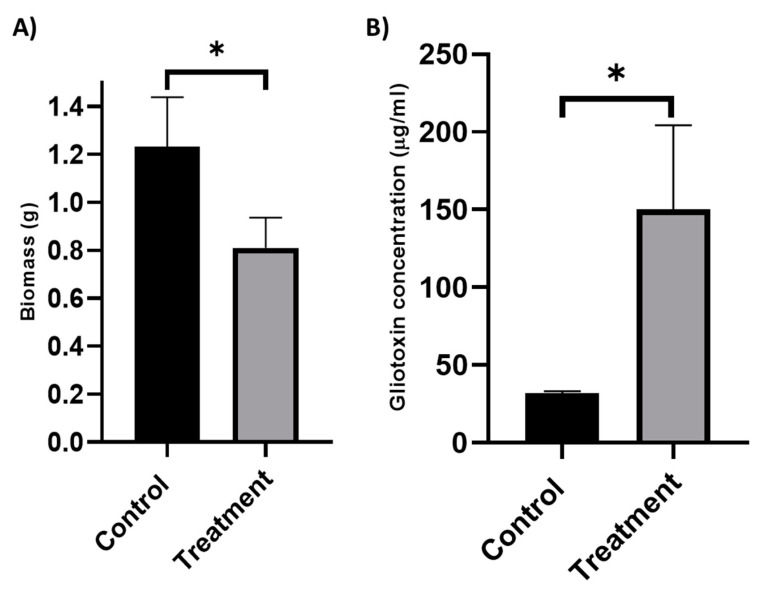
Analysis of the effects of *K. pneumoniae* culture filtrate on *A. fumigatus* mycelial growth (**A**) and Gliotoxin production (**B**) *K. pneumoniae* culture filtrate (25% *v*/*v*) or PBS (control) was added to 48 h old *A. fumigatus* and wet weight were recorded after 24 h growth (n = 3). Error bars represent SD. * *p* = 0.03 (**B**) Gliotoxin concentration was assessed by Reverse-Phase HPLC (n = 3), and error bars represent SD. * *p* = 0.01.

Exposure of 48 h old *A. fumigatus* to *K. pneumoniae* culture filtrate (25% *v*/*v*) for 24 h led to a decrease in fungal biomass (1.23 ± 0.20 g vs. 0.81± 0.12 g *p* = 0.03) (Figure 1A) and a large increase in gliotoxin secretion with a 371.55% increase (*p* = 0.01) (Figure 1B).

### 3.2. Characterisation of K. pneumoniae Culture Filtrate

Qualitative proteomic analysis of *K. pneumoniae* culture filtrate revealed the presence of 160 high-confidence proteins (Table S1), of which 35 were identified as having possible antifungal effects (Table 1). These could be subdivided into six categories; structurally bound (3 proteins), membrane-associated proteins (10 proteins), virulence-associated proteins (3 proteins), proteins with enzymatic activity (8 proteins), metal binding proteins (5 proteins) and proteins with detoxification activity (6 proteins).

### 3.3. Analysis of the Effect of K. pneumoniae Culture Filtrate on the A. fumigatus Proteome

**Figure 2:**
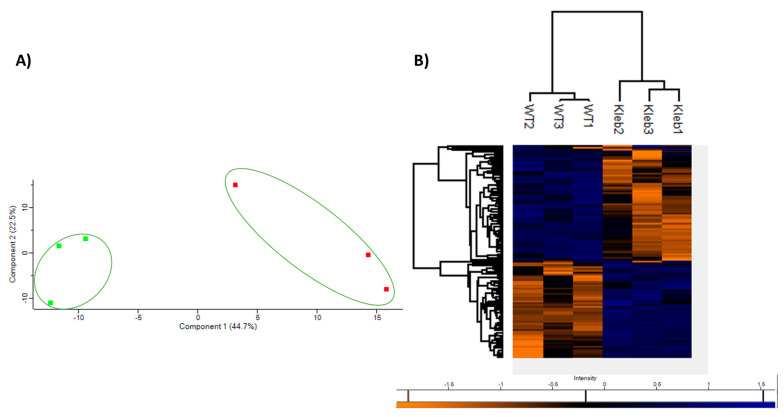
(**A**) Principle component analysis of *A. fumigatus* after exposure to *K. pneumoniae* culture filtrate (25% *v*/*v*) (green) and control *A. fumigatus* (red). (**B**) Heatmap generated through Two-way unsupervised hierarchical clustering of the median protein expression values of all statistically significant differentially abundant proteins.

Label-free mass spectrometry was employed to characterise the proteomic response of *A. fumigatus* following exposure to *K. pneumoniae* culture filtrate for 24 h. In total, 1960 proteins were identified, and 111 proteins were identified as being statistically significant (*p* < 0.05) differentially abundant (SSDA), having a fold change greater than ±1.5 (Table S2). A principle component analysis (PCA) was conducted on all significant proteins to identify distinct proteomic differences between each of the groups. The PCA indicates that the *A. fumigatus* exposed to the *K. pneumoniae* culture filtrate displays a distinct proteomic pattern when compared to control samples (Figure 2A). Hierarchal clustering carried out in Perseus highlights the clear differences in protein abundance between the control and the *A. fumigatus* culture that was exposed to the *K. pneumoniae* culture filtrate. These differences in protein abundance are highlighted in a heat map (Figure 2B), with blue indicating proteins with increased abundance and orange indicating proteins with decreased abundance, respectively.

**Figure 3:**
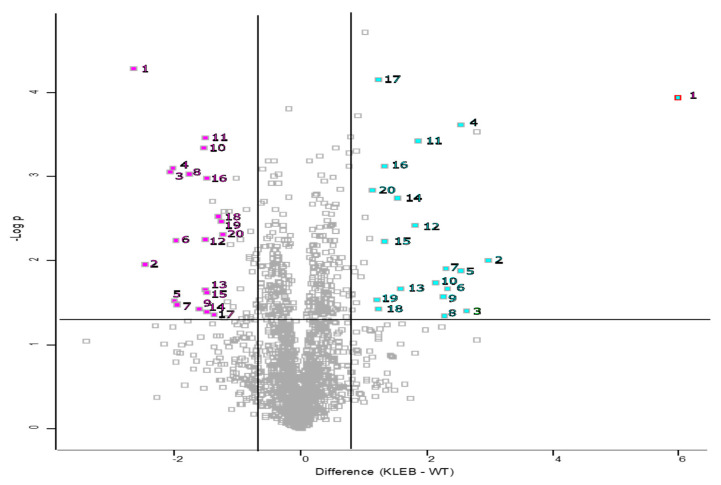
Volcano plot showing the distribution of statistically significant and differentially abundant (SSDA) proteins which have a −log (*p*-value) > 1.3 and difference +/−0.58. *A. fumigatus* exposed to *K. pneumoniae* culture filtrate compared to control *A. fumigatus*.

A volcano plot was generated by way of pairwise t-tests (*p* < 0.05) to determine the proteins which increased and decreased in abundance between control *A. fumigatus* samples and *A. fumigatus* exposed to *K. pneumoniae* culture filtrate (Figure 3). When analysing samples of *A. fumigatus* exposed to the *K. pneumoniae* culture filtrate, a significant increase in the relative abundance of proteins associated with secondary metabolism, in particular, proteins associated with gliotoxin biosynthesis was observed (Table 2), i.e., glutathione S-transferase GliG (+6.17 fold increase), non-ribosomal peptide synthase GliP (+3.67 fold), O-methyltransferase GliM (+3.5 fold), gamma-glutamyl acyltransferase GliK (+2.89 fold) and thioredoxin reductase GliT (+2.33 fold). In addition, ribonuclease mitogillin was increased in abundance by +4.9 fold. Fibrinogen C-terminal domain-containing protein and polysaccharide deacetylase family protein were increased by +63.3 fold and +6.9 fold, respectively, and both have been implemented in adherence to the lung epithelium. Decreased abundance of methyl sterol monooxygenase erg25B (−2.9 fold) and calcium/calmodulin-dependent protein kinase (−4.2 fold) indicates a decrease in fungal cell division (Table 3). Deoxyribose-phosphate aldolase was decreased in abundance by −2.3 fold and played a role in gluconeogenesis and lipid biogenesis. Protein synthesis was also affected by a decrease in the abundance of the 60 S ribosomal protein L22 and aspartyl aminopeptidase (−2.9 fold).

## 4. Discussion

The aim of the work presented here was to characterise the response of *A. fumigatus* to *K. pneumoniae* culture filtrate, as this might give an indication of the in vivo interaction between the fungus and bacteria. The culture conditions were designed to represent conditions within the immunocompromised lung by exposing *A. fumigatus* to the *K. pneumoniae* culture filtrate as opposed to bacterial cells. Czapek–Dox was chosen for this investigation as this medium provides both a low-nutrient and high-nitrogen environment, which are characteristics of immunocompromised lungs [19,20].

The results demonstrated that exposure to the *K. pneumoniae* culture filtrate inhibited the growth of *A. fumigatus* and induced increased secretion of gliotoxin. Previously, an examination of the interaction between *K. pneumoniae* cells and *A. fumigatus* demonstrated a reduction in spore germination and hyphal development which was dependent upon direct physical interaction between bacterial and fungal cells [17]. Exposure of *Penicillium verrucosum* to actinobacter-species cell-free supernatant also resulted in stimulation of ochratoxin A production and inhibition of fungal growth [21]. *Aspergillus flavus* exposed to culture filtrate of *Streptomyces* spp. also showed reduced growth and elevated secretion of aflatoxin B1 [22].

Analysis of the *K. pneumoniae* culture filtrate identified 160 high-confidence proteins (Table S1), of which 35 were identified to have possible effects on fungal development (Table 1) and three of which (putative lipoprotein, outer membrane protein A and chaperone protein DnaK) were also bound to the fungal mycelia. Outer membrane protein A is essential for *A. baumannii* cell attachment to *Candida albicans* filaments and A549 human alveolar epithelial cells [23] and has been demonstrated to induce leaky barriers in murine lungs and human A549 cells without affecting cell viability [24]. Elongation factor Tu was identified in the *K. pneumoniae* culture filtrate is involved in bacterial virulence and is associated with adhesion to host extracellular matrix components. Secretion of EF-Tu increased after *Heliobacter pylori* infection, suggesting that *H. pylori* secrete EF-Tu to facilitate attachment to host cells [25].

The ability of the *K. pneumoniae* culture filtrate to inhibit fungal growth could have also arisen as a result of enzymatic activity. DegP-like periplasmic serine endoprotease is a highly conserved periplasmic protease found in most Gram-negative bacteria. This protease is involved in the degradation of denatured or aggregated protein within the cell envelope in *E coli* [26,27]. Protease VII (OmpT), an aspartic protease, was identified in the culture filtrate and is associated with the inhibition of coagulation and antimicrobial peptide production [28].

Redox-active components and enzymes in the *K. pneumoniae* culture filtrate could also affect fungal growth and mycotoxin production. Thiol:disulfide interchange protein DsbA plays a role in oxidising the formation of disulfide bonds [29]. Thiol:disulfide interchange protein DsbA may be implemented in the oxidation of gliotoxin’s disulfide bond, increasing its biological accumulation and activity [30]. Alkyl hydroperoxide reductase C was identified in the *K. pneumoniae* culture filtrate and catalyses the reduction of hydrogen peroxide, organic hydroperoxides and thioredoxin in HeLa cells [31].

The non-siderophore iron uptake component EfeO was identified in the *K. pneumoniae* culture filtrate, and this is a component in the main ferrous iron transport system that is present in both pathogenic and non-pathogenic microbes [32]. In addition, the high-affinity zinc uptake system protein ZnuA in *K. pneumoniae* culture filtrate is essential in acquiring zinc at the interface between bacteria and mammalian cells and counteracting host depletion mechanisms [33]. It has been previously demonstrated that zinc limitation results in increased gliotoxin production and the growth-limiting effects of exogenous gliotoxin are relieved by the presence of zinc in media [34]. In addition, certain genes of the gliotoxin biosynthetic cluster, including *gliZ*, are regulated by ZafA, which is the zinc-responsive transcription factor that controls the adaptive response to zinc starvation in *A. fumigatus* [35]. Analysis of *P. aeruginosa* culture filtrate, which also affected fungal growth and secondary metabolism, identified a similar protein profile to that found in this work [14]. *P. aeruginosa* culture filtrate contained chaperone protein DnaK and components involved in the uptake of nutrients, including ferric iron-binding periplasmic protein HitA. Detoxification proteins such as thiol:disulphide interchange protein DsbA and thioredoxin reductase were found in both culture filtrates. Despite these similarities, *K. pneumoniae* culture filtrate promoted gliotoxin biosynthesis, while *P. aeruginosa* inhibited it.

*A. fumigatus* exposed to the *K. pneumoniae* culture filtrate demonstrated a reduction in growth and a shift towards secondary metabolism, particularly through the increased secretion of gliotoxin. Gliotoxin biosynthesis is mediated by a gene cluster consisting of 13 genes. Proteomic analysis revealed that five proteins involved in the biosynthesis of gliotoxin were increased in abundance; glutathione S-transferase (+6.17 fold), non-ribosomal peptide synthase (+3.67 fold), O-methyltransferase (+3.5 fold), gamma-glutamyl acyltransferase (+2.89 fold) and thioredoxin reductase (+2.33 fold). The increase in gliotoxin production may be attributed to the physical binding of outer membrane protein A, due to its ability to induce leakage in epithelial cells [24] in a similar manner to fungistatic concentrations of amphotericin B which increases *A. fumigatus* permeability and stimulates *de novo* gliotoxin biosynthesis [36].

There was also an increase (+63.3 fold) in the abundance of fibrinogen C-terminal domain-containing protein, and this could indicate increased virulence and adhesion to lung epithelium. Asthmatic lungs have damaged bronchioloalveolar epithelium, and fibrinogen deposits form at the surface of wounded epithelia, which facilitate microbial attachment [37]. *A. fumigatus* also demonstrated increased binding affinity to fibrinogen compared with less pathogenic *Aspergillus spp.,* suggesting that adhesion to the extracellular matrix may be important in disease pathogenesis [38]. Polysaccharide deacetylase family protein was increased in abundance (+6.9 fold) and is associated with adherence as proteins with similar domains, such as Agd3, are part of a group of metal-dependent polysaccharide deacetylases, which have been shown to remove *N*- or *O*-linked acetate groups from chitin, peptidoglycan, acetylxylan, and poly-β-1,6-*N*-acetylglucosamine, deletion of *agd3* was associated with a reduced adherence and virulence in murine models of invasive aspergillosis, indicating reduced fungal burden [39].

The proteome of *A. fumigatus* exposed to *K. pneumoniae* culture filtrate also showed a reduced abundance of several proteins; 1,3 beta glucanosyltransferase was reduced in abundance by −3.9 fold and plays a role in cell wall biogenesis [40]. The membrane protein methyl sterol monooxygenase erg25B, essential for ergosterol biosynthesis [41], was reduced by −2.9 fold, calcium/calmodulin-dependent protein kinase was reduced by −4.2 fold and is essential for fungal nuclear cell division and hyphal development in *Aspergillus nidulans* [42]. Deoxyribose-phosphate aldolase was reduced in abundance by −2.3 fold and had a role in gluconeogenesis and the glyoxylate cycle [43]. Decreased abundance of methyltransferase LaeA-like putative protein −2.16 fold could explain the downregulation of proteins associated with specific secondary metabolites [44], including helvolic acid and fumagillin. Proteins associated with helvolic acid biosynthesis, including cytochrome P450 monooxygenase helB1 (−2.8 fold), short chain dehydrogenase helC (−3.8 fold), protostadienol synthase helA (−2.8 fold) and 3-ketosteroid 1-dehydrogenase helE (−2.4 fold) were decreased in abundance and the fumagillin associated protein polyketide transferase af380 (−3.8 fold) was also decreased in abundance [45,46].

This study provides evidence that exposure of *A. fumigatus* to *K. pneumoniae* culture filtrate results in a reduction in growth but an increase in gliotoxin production and in the abundance of associated proteins. The clinical implications of these alterations could inhibit the ability of the patient to mount an effective immune response to *A. fumigatus* infection resulting in more severe symptoms and an increase in mortality. This work provides novel insights into an important bacterial-fungal interaction that occurs in the lungs of immunocompromised patients. Understanding the extent and importance of such microbial interactions can help to identify better therapeutic strategies for the control of pulmonary infections in susceptible patients.

## Figures and Tables

**Table 1:** Proteins identified in *K. pneumoniae* culture filtrate with potential antifungal activity.

Protein Name	Uniprot Code	Unique Peptides	% Coverage	Function
Outer membrane protein A	B5XY48	8	22.28	Membrane proteins
Outer membrane protein X	B5XYT0	7	39.76	
Outer membrane protein C	B5XNZ9	7	21.48	
Penicillin-binding protein activator LpoA	B5XSZ6	5	9.54	
OmpA family protein	B5XN00	3	24.54	
Penicillin-binding protein activator LpoB	B5XXG6	2	16.74	
OmpA family protein	B5XVK9	3	28.75	
LPS-assembly protein LptD	B5Y1Z1	3	5.37	
MrkF protein	B5XUK5	2	16.33	
LPS-assembly lipoprotein LptE	B5XZR3	2	13.26	
Elongation factor Tu	B5XN88	10	40.35	virulence
Elongation factor Ts	B5Y1K1	2	12.01	
Tol-Pal system protein TolB	B5XZC1	6	24.18	
Pectinesterase	B5XZ84	7	22.01	Enzymatic activity
Autonomous glycyl radical cofactor	B5XNF9	6	51.18	
Protease VII	B5RKF2	6	25.72	
Periplasmic serine endoprotease DegP-like	B5Y1K8	6	15.93	
Enolase	B5XV19	6	21.06	
Endolytic peptidoglycan transglycosylase RlpA	B5XZS1	5	15.06	
Beta-lactamase	B5XQY6	4	11.88	
Prephenate dehydratase/arogenate dehydratase	B5XVG4	3	16.60	
Metal-binding protein	B5XZ21	6	39.35	Metal binding
High-affinity zinc uptake system protein ZnuA	B5XQ08	5	25.47	
Copper homeostasis protein CutF	B5Y1H9	3	16.81	
Iron uptake system component EfeO	B5XXM1	3	10.4	
Thiol:disulfide interchange protein	B5XZJ6	4	31.40	
Alkyl hydroperoxide reductase C	B5XZT7	4	35.82	Detoxification
Thioredoxin	B5XYY8	5	51.37	
Acriflavine resistance protein A	B5Y0P5	3	9.57	
Thiol peroxidase	B5XRV9	3	32.14	
Alkyl hydroperoxide reductase C	B5Y0Z0	3	35.82	
Glutathione ABC transporter, periplasmic glutathione-binding protein	B5XYQ7	2	2.92	
Inhibitor of vertebrate lysozyme	B5Y004	2	15.54	
Putative lipoprotein	B5XUP5	1	36.1	Structurally bound
Outer membrane protein A	B5XY48	6	19.1	
Chaperone protein DnaK	B5Y242	2	2.7	

**Table 2:** The Top 20 proteins most increased in abundance in *A. fumigatus* after exposure to *K. pneumoniae* culture filtrate for 24 h.

Number	Fold Change	Protein Name	Protein IDs	Unique Peptides	Sequence Coverage [%]
1	63.39	Fibrinogen C-terminal domain-containing protein	Q4W8X0	3	38.5
2	7.88	SGL domain-containing protein	Q4WP91	4	15
3	6.93	Polysaccharide deacetylase family protein	Q4WUN9	9	48.7
4	6.17	Glutathione S-transferase gliG	A4GYZ0	18	73.3
5	5.81	ABM domain-containing protein	Q4WG08	2	24.3
6	5.80	Endonuclease/exonuclease/phosphatase family	Q4WKR6	8	31.9
7	5.04	D-xylose reductase (NAD(P)H)	Q4WI64	10	38.6
8	4.96	Ribonuclease mitogillin	P67875	6	44.9
9	4.86	Amine oxidase	Q4WFX6	16	46
10	4.79	DUF4468 domain-containing protein	Q4WMI8	9	49.7
11	4.42	DUF907 domain protein	Q4WHA4	1	3
12	3.67	Nonribosomal peptide synthetase gliP	Q4WMJ7	14	14.2
13	3.51	O-methyltransferase gliM	Q4WMJ5	12	32.9
14	2.98	Oxidoreductase, short-chain	Q4WUP1	5	40.5
15	2.89	Gamma-glutamyl cyclotransferase gliK	E9R9Y3	2	16.8
16	2.50	GPI anchored serine-threonine rich protein	Q4WTF2	2	34.5
17	2.50	Phosphatidylglycerol/phosphatidylinositol transfer	Q4X136	11	46.9
18	2.35	Cache_2 domain-containing protein	Q4WYY2	4	50.8
19	2.34	Thioredoxin reductase gliT	E9RAH5	20	85.9
20	2.33	Cell wall protein PhiA	Q4WF87	6	73

**Table 3:** The top 20 proteins most decreased in abundance in *A. fumigatus* after exposure to *K. pneumoniae* culture filtrate for 24 h.

Number	Fold Change	Protein Name	Protein IDs	Unique Peptides	Sequence Coverage [%]
1	−6.30	HYPK_UBA domain-containing protein	Q4WPC3	2	17.7
2	−5.49	Methyltransferase	Q4X081	4	30.3
3	−4.20	Calcium/calmodulin dependent protein kinase	Q4WXH7	7	25.1
4	−4.05	Elongation of fatty acids protein	Q4WEE9	4	16
5	−3.97	1,3-beta-glucanosyltransferase	Q4WBF7	4	8.9
6	−3.96	Protein DOM34 homolog	Q4WI62	7	26.6
7	−3.86	Short chain dehydrogenase helC	Q4WR19	7	37.7
8	−3.84	Polyketide transferase af380	Q4WAY4	8	47.9
9	−3.42	BTB/POZ domain protein	Q4WFH8	6	32
10	−3.02	Methyltransferase psoC	Q4WB00	21	71.2
11	−2.86	Methylsterol monooxygenase erg25B	Q4W9I3	3	13.9
12	−2.86	DUF948 domain-containing protein	Q4WXM0	4	42.3
13	−2.85	Cytochrome P450 monooxygenase helB1	Q4WR17	9	20.5
14	−2.83	Aspartyl aminopeptidase	Q4WX56	5	23.4
15	−2.80	Protostadienol synthase helA	Q4WR16	14	28.2
16	−2.80	Signal recognition particle 54 kDa protein	Q4WEQ8	3	11.5
17	−2.79	Tripeptidyl-peptidase sed4	Q4WQU0	3	8.1
18	−2.64	60S ribosomal protein L22, putative	Q4WYA0	7	52.1
19	−2.58	Amino acid permease (Gap1), putative	Q4WG99	12	19
20	−2.46	3-ketosteroid 1-dehydrogenase helE	Q4WR24	6	18.3

## Data Availability

The mass spectrometry proteomics data have been deposited to the ProteomeXchange Consortium via the PRIDE [18] partner repository with the dataset identifier PXD037833.

## Supplementary Materials

The following supporting information can be downloaded at: https://www.mdpi.com/article/10.3390/jof9020222/s1, Table S1: All high-confidence proteins identified in *K. pneumoniae* culture filtrate. Table S2: All statistically significant differentially abundant proteins identified following exposure to *Klebsiella pneumoniae* cell-free culture filtrate at a concentration of 25% *v*/*v* for 24 h.

## References

[1] O’Dwyer D.N., Dickson R.P., Moore B.B. (2016). The Lung Microbiome, Immunity, and the Pathogenesis of Chronic Lung Disease. J. Immunol..

[2] Leitao Filho F.S., Alotaibi N.M., Ngan D., Tam S., Yang J., Hollander Z., Chen V., FitzGerald J.M., Nislow C., Leung J.M. (2019). Sputum Microbiome Is Associated with 1-Year Mortality after Chronic Obstructive Pulmonary Disease Hospitalizations. Am. J. Respir. Crit. Care Med..

[3] Keown K., Reid A., Moore J.E., Taggart C.C., Downey D.G. (2020). Coinfection with *Pseudomonas aeruginosa* and *Aspergillus fumigatus* in Cystic Fibrosis. Eur. Respir. Rev..

[4] LiPuma J.J. (2010). The Changing Microbial Epidemiology in Cystic Fibrosis. Clin. Microbiol. Rev..

[5] Navon-Venezia S., Kondratyeva K., Carattoli A. (2017). *Klebsiella pneumoniae*: A Major Worldwide Source and Shuttle for Antibiotic Resistance. FEMS Microbiol. Rev..

[6] Vieira A.T., Rocha V.M., Tavares L., Garcia C.C., Teixeira M.M., Oliveira S.C., Cassali G.D., Gamba C., Martins F.S., Nicoli J.R. (2016). Control of *Klebsiella pneumoniae* Pulmonary Infection and Immunomodulation by Oral Treatment with the Commensal Probiotic Bifidobacterium Longum 51A. Microbes Infect..

[7] Fodah R.A., Scott J.B., Tam H.-H., Yan P., Pfeffer T.L., Bundschuh R., Warawa J.M. (2014). Correlation of *Klebsiella pneumoniae* Comparative Genetic Analyses with Virulence Profiles in a Murine Respiratory Disease Model. PLoS ONE.

[8] Delfino E., Giacobbe D.R., Del Bono V., Coppo E., Marchese A., Manno G., Morelli P., Minicucci L., Viscoli C. (2015). First Report of Chronic Pulmonary Infection by KPC-3-Producing and Colistin-Resistant *Klebsiella pneumoniae* Sequence Type 258 (ST258) in an Adult Patient with Cystic Fibrosis. J. Clin. Microbiol..

[9] Lattanzi C., Messina G., Fainardi V., Tripodi M.C., Pisi G., Esposito S. (2020). Allergic Bronchopulmonary Aspergillosis in Children with Cystic Fibrosis: An Update on the Newest Diagnostic Tools and Therapeutic Approaches. Pathogens.

[10] Schweer K.E., Bangard C., Hekmat K., Cornely O.A. (2014). Chronic Pulmonary Aspergillosis. Mycoses.

[11] Kradin R.L., Mark E.J. (2008). The Pathology of Pulmonary Disorders Due to *Aspergillus* Spp.. Arch. Pathol. Lab. Med..

[12] Webb B.J., Ferraro J.P., Rea S., Kaufusi S., Goodman B.E., Spalding J. (2018). Epidemiology and Clinical Features of Invasive Fungal Infection in a US Health Care Network. Open Forum Infect. Dis..

[13] Bassetti M., Peghin M., Vena A. (2018). Challenges and Solution of Invasive Aspergillosis in Non-Neutropenic Patients: A Review. Infect. Dis. Ther..

[14] Margalit A., Sheehan D., Carolan J.C., Kavanagh K. (2022). Exposure to the *Pseudomonas aeruginosa* secretome alters the proteome and secondary metabolite production of *Aspergillus fumigatus*. Microbiology.

[15] Margalit A., Carolan J.C., Sheehan D., Kavanagh K. (2020). The *Aspergillus fumigatus* Secretome Alters the Proteome of *Pseudomonas aeruginosa* to Stimulate Bacterial Growth: Implications for Co-Infection. Mol. Cell. Proteom..

[16] Dees J., Connell J., Stacy A., Turner K., Whiteley M. (2014). Mechanisms of Synergy in Polymicrobial Infections. J. Microbiol. Seoul Korea.

[17] Nogueira M.F., Pereira L., Jenull S., Kuchler K., Lion T. (2019). *Klebsiella pneumoniae* Prevents Spore Germination and Hyphal Development of *Aspergillus* Species. Sci. Rep..

[18] Perez-Riverol Y., Bai J., Bandla C., García-Seisdedos D., Hewapathirana S., Kamatchinathan S., Kundu D.J., Prakash A., Frericks-Zipper A., Eisenacher M. (2021). The PRIDE Database Resources in 2022: A Hub for Mass Spectrometry-Based Proteomics Evidences. Nucleic Acids Res..

[19] Line L., Alhede M., Kolpen M., Kühl M., Ciofu O., Bjarnsholt T., Moser C., Toyofuku M., Nomura N., Høiby N. (2014). Physiological Levels of Nitrate Support Anoxic Growth by Denitrification of *Pseudomonas aeruginosa* at Growth Rates Reported in Cystic Fibrosis Lungs and Sputum. Front. Microbiol..

[20] Lu Z., Huang W., Wang L., Xu N., Ding Q., Cao C. (2018). Exhaled Nitric Oxide in Patients with Chronic Obstructive Pulmonary Disease: A Systematic Review and Meta-Analysis. Int. J. Chron. Obstruct. Pulmon. Dis..

[21] Campos-Avelar I., Colas de la Noue A., Durand N., Fay B., Martinez V., Fontana A., Strub C., Schorr-Galindo S. (2020). Minimizing Ochratoxin A Contamination through the Use of Actinobacteria and Their Active Molecules. Toxins.

[22] Campos-Avelar I., Colas de la Noue A., Durand N., Cazals G., Martinez V., Strub C., Fontana A., Schorr-Galindo S. (2021). *Aspergillus flavus* Growth Inhibition and Aflatoxin B1 Decontamination by Streptomyces Isolates and Their Metabolites. Toxins.

[23] Gaddy J.A., Tomaras A.P., Actis L.A. (2009). The Acinetobacter Baumannii 19606 OmpA Protein Plays a Role in Biofilm Formation on Abiotic Surfaces and in the Interaction of This Pathogen with Eukaryotic Cells. Infect. Immun..

[24] Zhang W., Zhou H., Jiang Y., He J., Yao Y., Wang J., Liu X., Leptihn S., Hua X., Yu Y. (2022). *Acinetobacter baumannii* Outer Membrane Protein A Induces Pulmonary Epithelial Barrier Dysfunction and Bacterial Translocation Through The TLR2/IQGAP1 Axis. Front. Immunol..

[25] Chiu K.-H., Wang L.-H., Tsai T.-T., Lei H.-Y., Liao P.-C. (2017). Secretomic Analysis of Host–Pathogen Interactions Reveals That Elongation Factor-Tu Is a Potential Adherence Factor of *Helicobacter pylori* during Pathogenesis. J. Proteome Res..

[26] Jones C.H., Bolken T.C., Jones K.F., Zeller G.O., Hruby D.E. (2001). Conserved DegP Protease in Gram-Positive Bacteria Is Essential for Thermal and Oxidative Tolerance and Full Virulence in *Streptococcus pyogenes*. Infect. Immun..

[27] Zhang S., Cheng Y., Ma J., Wang Y., Chang Z., Fu X. (2019). Degp Degrades a Wide Range of Substrate Proteins in *Escherichia coli* under Stress Conditions. Biochem. J..

[28] Sabotič J., Kos J. (2012). Microbial and Fungal Protease Inhibitors—Current and Potential Applications. Appl. Microbiol. Biotechnol..

[29] Denoncin K., Collet J.-F. (2013). Disulfide Bond Formation in the Bacterial Periplasm: Major Achievements and Challenges Ahead. Antioxid. Redox Signal..

[30] Bernardo P.H., Brasch N., Chai C.L.L., Waring P. (2003). A Novel Redox Mechanism for the Glutathione-Dependent Reversible Uptake of a Fungal Toxin in Cells. J. Biol. Chem..

[31] Choi H.S., Shim J.S., Kim J.-A., Kang S.W., Kwon H.J. (2007). Discovery of Gliotoxin as a New Small Molecule Targeting Thioredoxin Redox System. Biochem. Biophys. Res. Commun..

[32] Lau C.K.Y., Krewulak K.D., Vogel H.J. (2016). Bacterial Ferrous Iron Transport: The Feo System. FEMS Microbiol. Rev..

[33] Neupane D.P., Kumar S., Yukl E.T. (2019). Two ABC Transporters and a Periplasmic Metallochaperone Participate in Zinc Acquisition in *Paracoccus Denitrificans*. Biochemistry.

[34] Traynor A.M., Owens R.A., Coughlin C.M., Holton M.C., Jones G.W., Calera J.A., Doyle S. (2021). At the Metal-Metabolite Interface in *Aspergillus fumigatus*: Towards Untangling the Intersecting Roles of Zinc and Gliotoxin. Microbiol. Read. Engl..

[35] Vicentefranqueira R., Amich J., Marín L., Sánchez C.I., Leal F., Calera J.A. (2018). The Transcription Factor ZafA Regulates the Homeostatic and Adaptive Response to Zinc Starvation in *Aspergillus fumigatus*. Genes.

[36] Reeves E.P., Murphy T., Daly P., Kavanagh K. (2004). Amphotericin B Enhances the Synthesis and Release of the Immunosuppressive Agent Gliotoxin from the Pulmonary Pathogen *Aspergillus fumigatus*. J. Med. Microbiol..

[37] Upadhyay S.K., Gautam P., Pandit H., Singh Y., Basir S.F., Madan T. (2012). Identification of Fibrinogen-Binding Proteins of Aspergillus Fumigatus Using Proteomic Approach. Mycopathologia.

[38] Wasylnka J.A., Moore M.M. (2000). Adhesion of *Aspergillus* Species to Extracellular Matrix Proteins: Evidence for Involvement of Negatively Charged Carbohydrates on the Conidial Surface. Infect. Immun..

[39] Lee M.J., Geller A.M., Bamford N.C., Liu H., Gravelat F.N., Snarr B.D., Le Mauff F., Chabot J., Ralph B., Ostapska H. (2016). Deacetylation of Fungal Exopolysaccharide Mediates Adhesion and Biofilm Formation. mBio.

[40] Gastebois A., Fontaine T., Latgé J.-P., Mouyna I. (2010). β(1-3)Glucanosyltransferase Gel4p Is Essential for *Aspergillus fumigatus*. Eukaryot. Cell.

[41] Alcazar-Fuoli L., Mellado E., Garcia-Effron G., Lopez J.F., Grimalt J.O., Cuenca-Estrella J.M., Rodriguez-Tudela J.L. (2008). Ergosterol Biosynthesis Pathway in *Aspergillus fumigatus*. Steroids.

[42] Alcazar-Fuoli L., Mellado E. (2013). Ergosterol Biosynthesis in *Aspergillus fumigatus*: Its Relevance as an Antifungal Target and Role in Antifungal Drug Resistance. Front. Microbiol..

[43] Dayton J.S., Means A.R. (1996). Ca(2+)/Calmodulin-Dependent Kinase Is Essential for Both Growth and Nuclear Division in *Aspergillus Nidulans*. Mol. Biol. Cell.

[44] Vorapreeda T., Khongto B., Thammarongtham C., Srisuk T., Laoteng K. (2021). Metabolic Regulation of Sugar Assimilation for Lipid Production in *Aspergillus oryzae* BCC7051 through Comparative Transcriptome Perspective. Biology.

[45] Keller N., Bok J., Chung D., Perrin R.M., Keats Shwab E. (2006). LaeA, a Global Regulator of *Aspergillus* Toxins. Med. Mycol..

[46] Lin H.-C., Chooi Y.-H., Dhingra S., Xu W., Calvo A.M., Tang Y. (2013). The Fumagillin Biosynthetic Gene Cluster in *Aspergillus fumigatus* Encodes a Cryptic Terpene Cyclase Involved in the Formation of β-Trans-Bergamotene. J. Am. Chem. Soc..

