# Supplementary material for: Exposure of Aspergillus fumigatus to Klebsiella pneumoniae Culture Filtrate Inhibits Growth and Stimulates Gliotoxin Production
Source: J Fungi (Basel). 2023 Feb 8;9(2):222. doi: 10.3390/jof9020222 (PMC9961802; doi:10.3390/jof9020222)
Supplement: Supplementary file 1 [file jof-09-00222-s001.zip › jof-2185616-supplementary.pdf]

Table S1: All high confidence Proteins identified in *K. pneumoniae* culture filtrate

Protein name	Uniprot code	# Unique Peptides	Coverage [%]
Histidine ABC transporter, periplasmic histidine-binding protein	B5XNT0	15	65
Glutamate/aspartate ABC transporter, periplasmic glutamate/aspartate-binding protein	B5XZQ5	10	39.60
Molybdate ABC transporter, periplasmic molybdate-binding protein	B5XZA4	14	55.64
Lysine-arginine-ornithine-binding periplasmic protein	B5XNS8	14	53.46
Outer membrane protein A	B5XY48	9	25.42
Putative lipoprotein OS	B5XWW2	6	60.27
Glutamine ABC transporter, periplasmic glutamine-binding protein	B5XYT3	9	43.95
High-affinity branched-chain amino acid ABC transporter, periplasmic Leu/Ile/Val-binding protein	B5XTN1	10	43.32
Phosphate-binding protein PstS	B5XZM9	11	36.70
Outer membrane protein TolC	B5XU48	12	41.54
Elongation factor Tu	B5XN88	12	46.19
Outer membrane protein assembly factor BamB	B5XNL8	9	38.52
Tol-Pal system protein TolB	B5XZC1	9	40.93
Osmotically-inducible lipoprotein B	B5XS07	2	57.53
Lipoprotein	B5Y1H4	8	43.17
Autonomous glycyl radical cofactor	B5XNF9	9	74.01
Putative lipoprotein	B5XUP5	4	55.55
Branched-chain amino acid ABC transporter, periplasmic amino acid-binding protein	B5Y143	7	34.23
Metal-binding protein	B5XZ21	7	39.35
Osmotically-inducible lipoprotein E	B5XS39	4	37.5
Fimbrial protein	B5XUK8	6	22.35
Arginine ABC transporter, periplasmic arginine-binding protein ArtI	B5XYM1	6	30.86
Outer membrane lipoprotein SlyB	B5XWN6	5	47.09
Cation ABC transporter, periplasmic cation-binding protein	B5XVB4	6	23.97
Putative amino acid ABC transporter, periplasmic amino acid-binding protein	B5Y150	7	33.45
Outer membrane protein C	B5XNZ9	7	21.48
Outer membrane protein X OS	B5XYT0	6	39.76
RND multidrug efflux membrane fusion protein OqxA	B5XVH8	8	23.27
MltA-interacting protein MipA	B5XS75	5	30.24
Chaperone protein DnaK	B5Y242	7	21.15
Uncharacterized protein	B5XRD5	6	27.53
Cystine ABC transporter, periplasmic cystine-binding protein	B5XPW2	8	30.45
Arginine ABC transporter, periplasmic arginine-binding protein ArtJ	B5XYM4	6	30.45
Hydrophobic amino acid ABC transporter, periplasmic amino acid-binding protein	B5XW00	6	18.01

Dipeptide ABC transporter, periplasmic dipeptide-binding protein	B5XN08	8	22.61
High-affinity zinc uptake system protein ZnuA	B5XQ08	5	25.47
Peptidoglycan-associated protein	B5XZC0	5	44.82
Phospholipid-binding domain protein OS	B5XSZ3	5	36.64
Sulfate/thiosulfate ABC transporter, periplasmic thiosulfate-binding protein	B5XVR8	8	23.66
Outer membrane usher protein MrkC	B5XUK7	7	12.68
Putative lipoprotein	B5Y0U6	6	57.29
Protease VII	B5RKF2	6	26.04
Pectinesterase	B5XZ84	6	18.26
Urea ABC transporter, urea binding protein	B5XQX4	4	14.89
Putrescine-binding periplasmic protein	B5XYN2	6	26.21
Inner membrane lipoprotein DcrB	B5XTL6	5	42.16
Periplasmic serine endoprotease DegP-like	B5Y1K8	6	17.61
Enolase	B5XV19	6	21.06
Outer membrane protein assembly factor BamC	B5XVM8	5	26.45
Glyceraldehyde-3-phosphate dehydrogenase	B5XS72	5	21.14
Penicillin-binding protein activator LpoA	B5XSZ6	7	14.67
Alkyl hydroperoxide reductase C	B5XZT7	6	44.38
Phospholipid-binding domain protein	B5XVC0	3	37.5
Thiol:disulfide interchange protein	B5XZJ6	4	31.40
Putative lipoprotein	B5XYL9	6	50.29
Sucrose porin ScrY	B5Y085	6	20.39
Outer membrane protein assembly factor BamA	B5Y1J4	6	12.73
YbiS protein	B5XYS1	6	28.28
High-affinity branched-chain amino acid ABC transporter, periplasmic leucine-specific-binding protein	B5XTN6	6	17.34
Chaperone protein Skp	B5Y1J3	4	31.67
YfaZ family protein	B5Y2E2	4	45.30
MrkF protein	B5XUK5	3	31.68
ABC transporter, quaternary amine uptake transporter (QAT) family, substrate-binding protein	B5XP81	5	31.14
Alkaline phosphatase	B5Y118	5	13.65
Co-chaperonin GroES	B5Y369	4	60.82
Amino acid ABC transporter, periplasmic amino acid-binding protein	B5XVH0	3	19.10
Peptidyl-prolyl cis-trans isomerase	B5XN80	4	25
Protein YcfR OS	B5XSQ4	3	43.67
Peptidase, M48B family	B5XUB5	3	20.23
Osmotically-inducible protein Y	B5Y281	4	28.15
Heat shock protein HslJ	B5XRM6	2	27.39
Toluene tolerance protein Ttg2D	B5XSU9	5	19.43
Uncharacterized protein	B5XQC4	4	18.25
Glucose-specific phosphotransferase enzyme IIA component	B5XVS7	4	36.09
OmpA family protein	B5XVK9	4	28.75
Putative lipoprotein	B5XVR5	4	24.08

Sugar ABC transporter, periplasmic sugar-binding protein	B5XZU7	4	17.88
Diacetyl reductase ((S)-acetoin forming)	B5XWD9	4	22.65
Chaperonin GroEL	B5Y368	4	11.31
Putative lipoprotein	B5XWS8	4	32.88
Endolytic peptidoglycan transglycosylase RlpA	B5XZS1	4	14.54
Phosphoglycerate kinase	B5XUB8	4	17.31
30S ribosomal protein S10	B5XN93	4	34.95
Thioredoxin	B5XYY8	2	32.11
Outer membrane lipoprotein RcsF	B5Y1H5	2	20.74
Elongation factor Ts	B5Y1K1	3	18.37
Putrescine-binding periplasmic protein	B5XSP6	3	13.50
Major outer membrane lipoprotein Lpp	B5XQH5	2	33.33
Oligopeptide ABC transporter, periplasmic oligopeptide-binding protein	B5XQA2	4	10.66
OmpA family protein	B5XN00	2	17.72
Gram-negative pili assembly chaperone	B5XUK6	3	21.16
Transketolase	B5XUB6	3	6.33
ABC transporter, periplasmic substrate-binding protein	B5Y015	3	9.96
Uncharacterized protein	B5XT34	3	9.43
Phosphocarrier protein HPr	B5XVS9	3	70.58
Oligopeptide/dipeptide ABC transporter, periplasmic oligopeptide/dipeptide-binding protein	B5Y034	3	7.20
Outer-membrane lipoprotein carrier protein	B5XYA1	3	17.73
Phosphonate ABC transporter, periplasmic phosphonate-binding protein	B5Y161	3	17.20
DNA-binding protein HU, alpha subunit	B5XYD8	3	31.11
Thiol peroxidase	B5XRV9	3	23.21
Penicillin-binding protein activator LpoB	B5XXG6	2	16.74
Protein RecA	B5XVB6	3	11.93
Amino acid ABC transporter, periplasmic amino acid-binding protein	B5Y072	2	12.79
DUF1471 domain-containing protein	B5Y0A7	2	24.44
Cysteine synthase	B5XVT0	3	12.38
Tat (Twin-arginine translocation) pathway signal sequence domain/peptidase M15 family protein	B5XY71	3	20.76
Porin D	B5XUB3	2	9.71
Uncharacterized protein	B5XQ29	2	25.89
50S ribosomal protein L9	B5Y305	3	22.81
Putative lipoprotein	B5XXC9	2	14.37
Beta-lactamase	B5XQY6	3	11.88
Vitamin B12 transporter BtuB	B5XZ10	3	7.92
Outer-membrane lipoprotein LolB	B5XW49	3	17.73
50S ribosomal protein L10	B5XYF7	3	21.81
50S ribosomal protein L11	B5XYF9	2	16.19
Glutathione ABC transporter, periplasmic glutathione-binding protein	B5XYQ7	3	5.45
Uncharacterized protein	B5XXP1	2	52

Copper homeostasis protein CutF	B5Y1H9	2	12.06
Prephenate dehydratase/arogenate dehydratase	B5XVG4	3	16.60
Peptidyl-prolyl cis-trans isomerase	B5XN78	3	17.17
NAD(P)H dehydrogenase (quinone)	B5XXP0	3	26.76
Alkyl hydroperoxide reductase C	B5Y0Z0	3	19.5
30S ribosomal protein S1	B5XY85	2	6.64
Outer membrane protein assembly factor BamD	B5XVL8	3	19.18
Serine hydroxymethyltransferase	B5XNI6	2	6.71
50S ribosomal protein L2	B5XN97	3	12.82
50S ribosomal protein L5	B5XNA5	2	14.52
Lipoprotein	B5XQD8	2	11.65
Uncharacterized protein	B5XP92	2	35.23
Trigger factor	B5Y0U3	2	6.48
50S ribosomal protein L7/L12	B5XYF6	2	19.00
50S ribosomal protein L6	B5XNA8	2	12.99
N-acetylmuramoyl-L-alanine amidase	B5Y335	2	7.76
Chaperone SurA	B5Y1Z2	2	7.00
Sigma-E factor regulatory protein RseB	B5XNG6	2	7.54
Thiamine-binding periplasmic protein	B5Y1X5	2	6.72
Cell division coordinator CpoB	B5XZB9	2	4.88
Putative lipoprotein	B5XWN8	2	25.86
UDP-sugar hydrolase/5'-nucleotidase	B5Y0M8	2	4.54
DUF1471 domain-containing protein	B5XMT0	2	47.05
Iron uptake system component EfeO	B5XXM1	2	9.06
DNA-directed RNA polymerase subunit omega	B5XTE8	2	14.28
Cell division protein DamX	B5XTU8	2	7.92
LPS-assembly lipoprotein LptE	B5XZR3	2	13.26
30S ribosomal protein S8	B5XNA7	2	20.76
Peptidyl-prolyl cis-trans isomerase	B5XN63	2	12.16
Succinate--CoA ligase [ADP-forming] subunit alpha	B5XZD0	2	6.92
BOF domain-containing protein	B5XU58	2	20
Periplasmic murein peptide-binding protein	B5XRV5	2	4.27
Uncharacterized protein	B5XNI2	2	10.52
Single-stranded DNA-binding protein	B5XXX3	2	13.71
D-galactose-binding periplasmic protein	B5XP60	2	8.43
Serine-type D-Ala-D-Ala carboxypeptidase	B5XZS2	2	6.26
50S ribosomal protein L1	B5XYF8	2	10.68
Ribose ABC transporter, periplasmic D-ribose-binding protein OS	B5XZK5	2	8.44
50S ribosomal protein L24	B5XNA4	2	22.11
50S ribosomal protein L30	B5XNB1	2	33.89
Glyceraldehyde-3-phosphate dehydrogenase	B5XRG0	2	7.53
ABC transporter, periplasmic substrate-binding protein	B5XWU1	2	7.08

Table S2: All statistically significant differentially abundant proteins identified following exposure to Klebsiella Pneumoniae cell free culture filtrate at a concentration of 25% v/v for 24 hours

Fold Change	Protein name	Protein IDs	Unique peptides	Sequence coverage [%]
63.39	Fibrinogen C-terminal domain-containing protein	Q4W8X0	3	38.5
7.88	SGL domain-containing protein	Q4WP91	4	15
6.93	Polysaccharide deacetylase family protein	Q4WUN9	9	48.7
6.17	Glutathione S-transferase gliG	A4GYZ0	18	73.3
5.81	ABM domain-containing protein	Q4WG08	2	24.3
5.80	Endonuclease/exonuclease/phosphatase family	Q4WKR6	8	31.9
5.04	D-xylose reductase (NAD(P)H)	Q4WI64	10	38.6
4.96	Ribonuclease mitogillin	P67875	6	44.9
4.86	Amine oxidase	Q4WFX6	16	46
4.79	DUF4468 domain-containing protein	Q4WMI8	9	49.7
4.42	DUF907 domain protein	Q4WHA4	1	3
3.67	Nonribosomal peptide synthetase gliP	Q4WMJ7	14	14.2
3.51	O-methyltransferase gliM	Q4WMJ5	12	32.9
2.98	Oxidoreductase, short-chain	Q4WUP1	5	40.5
2.89	Gamma-glutamyl cyclotransferase gliK	E9R9Y3	2	16.8
2.50	GPI anchored serine-threonine rich protein	Q4WTF2	2	34.5
2.50	Phosphatidylglycerol/phosphatidylinositol transfer	Q4X136	11	46.9
2.35	Cache_2 domain-containing protein	Q4WYY2	4	50.8
2.34	Thioredoxin reductase gliT	E9RAH5	20	85.9
2.33	Cell wall protein PhiA	Q4WF87	6	73
2.19	Allergen Asp f 4	O60024	14	51.2
2.13	Probable beta-glucosidase btgE	Q4WC60	7	20.2
2.03	Glutamyl-tRNA(Gln) amidotransferase, subunit A	Q4WGZ8	14	45.6
2.03	Mitochondrial enoyl reductase, putative	Q4WF62	19	60
1.99	Translation elongation factor eEF-1B gamma subunit, putative	Q4WR90	10	57.3
1.95	GNAT family acetyltransferase, putative	Q4WIV0	4	18.6
1.89	alpha-galactosidase	Q4WX16	4	23.6
1.85	Extracellular serine-rich protein, putative	Q4WX15	17	38.7
1.85	Trimethyllysine dioxygenase TmlH, putative	Q4WJF3	2	12.8
1.83	Pre-mRNA-processing protein 45	Q4WEH7	7	26.3
1.80	chitinase	Q4WY00	12	45.3
1.76	Alpha-galactosidase	Q4WCV4	14	47.9
1.76	Armadillo-type protein	Q4WDA7	5	3.4
1.75	Beta-fructofuranosidase, putative	Q4WIN3	16	33.3
1.72	Cytochrome b5, putative	Q4X246	3	38.1
1.72	Multiprotein-bridging factor 1	Q4WX89	3	16.2
1.71	Tripeptidyl-peptidase sed2	Q70J59	20	49
1.71	Mannitol 2-dehydrogenase	Q4WQY4	12	38.6

1.66	Aminotransferase family protein (LolT), putative	A4D9B0	20	63.8
1.65	Major allergen Asp f 2	P79017	13	50.6
1.65	GST N-terminal domain-containing protein	A4D9U4	8	51.1
1.60	Probable glucan endo-1,3-beta-glucosidase eglC	Q4WSV9	21	58.4
1.60	Thioredoxin reductase, putative	Q4WEM5	13	38.3
1.57	1,3-beta-glucanosyltransferase gel3	P0C955	11	35.7
1.54	Putative transferase caf17, mitochondrial	Q4WVK5	11	41.8
1.54	Nucleoporin NUP49/NSP49, putative	Q4WM91	7	20.8
1.53	Methyltransferase, putative	Q4WYI3	13	54.2
1.51	AAA_5 domain-containing protein	Q4WUS8	7	64
1.51	Arginine biosynthesis bifunctional protein ArgJ, mitochondrial	Q4WUE0	13	43.1
1.50	Beta-hexosaminidase	Q4WCB5	17	40
1.49	Probable glycosidase crf1	Q8J0P4	11	49.6
-1.49	Cleavage and polyadenylation specific factor 5	Q4WE76	8	32.6
-1.50	Translocation protein SEC62	Q4X0R3	5	18.2
-1.50	NMT1 domain-containing protein	Q4WEM3	12	58.2
-1.51	Aminotransferase, class III	Q4WRC1	18	65.5
-1.53	Bactericidal permeability-increasing protein	Q4X1Z0	45	59.4
-1.53	Oxidoreductase, FAD-binding, putative	Q4X073	10	45.3
-1.54	Carbonic anhydrase	A4DA32	7	48.7
-1.54	Uracil phosphoribosyltransferase	Q4WZP7	12	67.4
-1.55	COP9 signalosome complex subunit 6	Q4WU56	9	48.9
-1.55	ThiJ/PfpI family protein	Q4WE16	6	45.8
-1.56	U6 snRNA-associated Sm-like protein LSm1	Q4X0Z1	1	9
-1.57	HAD superfamily hydrolase, putative	Q4WUF5	16	60.2
-1.58	Peptidase inhibitor I78 family protein	Q4WTB4	6	85.2
-1.59	Skin secretory protein xP2-like	Q4WMQ2	3	21.6
-1.60	FAD-binding FR-type domain-containing protein	Q4WQG1	8	27
-1.60	Lipase, putative	Q4WEP8	11	50.3
-1.60	Conserved glutamic acid-rich protein	Q4WYE9	2	12.7
-1.61	Short-chain dehydrogenase/reductase	Q4WKX1	18	85.4
-1.66	2-oxoglutarate-dependent dioxygenase	Q4WKX0	26	67
-1.70	GNAT family acetyltransferase, putative	Q4WDU8	20	85.5
-1.72	adenine phosphoribosyltransferase	Q4WAS3	6	55.3
-1.72	Importin beta-5 subunit, putative	Q4WZT1	20	29.3
-1.73	Cyclin dependent kinase inhibitor Pho81, putative	Q4WNK0	6	10.8
-1.76	Inhibitor I9 domain-containing protein	Q4X0G1	6	60.5
-1.76	Thioredoxin, putative	Q4WB53	8	76.7
-1.81	Sterol 14-alpha demethylase cyp51B	E9QY26	14	39.9
-1.81	TLDc domain-containing protein	Q4WQV9	6	13
-1.82	Deoxyhypusine hydroxylase	Q4WHG5	4	17.9
-1.89	Uncharacterized protein	Q4WYH8	4	14
-1.92	DUF2236 domain-containing protein	Q4WT15	5	22.9
-1.92	Phosphatidylinositol transporter, putative	Q4WUQ1	3	9.8

-2.01	pH-response transcription factor pacC/RIM101	Q4WY67	3	7.4
-2.08	O-methylsterigmatocystin oxidoreductase, putative	Q4WA42	17	44.6
-2.16	Methyltransferase LaeA-like, putative	Q4WBD7	12	45.2
-2.21	Ubiquitin fusion degradation protein Ufd1, putative	Q4WKH7	7	28.4
-2.25	Orotidine 5'-phosphate decarboxylase	O13410	7	35.3
-2.29	Ubiquitin conjugating enzyme (UbcM), putative	Q4WCU1	7	73.8
-2.31	LipA and NB-ARC domain protein	Q4WML7	11	17.2
-2.35	6-hydroxytryprostatin B O-methyltransferase	Q4WAW6	14	48.5
-2.38	deoxyribose-phosphate aldolase	Q4WGW8	5	24.9
-2.46	3-ketosteroid 1-dehydrogenase helE	Q4WR24	6	18.3
-2.58	Amino acid permease (Gap1), putative	Q4WG99	12	19
-2.64	60S ribosomal protein L22, putative	Q4WYA0	7	52.1
-2.79	Tripeptidyl-peptidase sed4	Q4WQU0	3	8.1
-2.80	Signal recognition particle 54 kDa protein	Q4WEQ8	3	11.5
-2.80	Protostadienol synthase helA	Q4WR16	14	28.2
-2.83	Aspartyl aminopeptidase	Q4WX56	5	23.4
-2.85	Cytochrome P450 monooxygenase helB1	Q4WR17	9	20.5
-2.86	DUF948 domain-containing protein	Q4WXM0	4	42.3
-2.86	Methylsterol monooxygenase erg25B	Q4W9I3	3	13.9
-3.02	Methyltransferase psoC	Q4WB00	21	71.2
-3.42	BTB/POZ domain protein	Q4WFH8	6	32
-3.84	Polyketide transferase af380	Q4WAY4	8	47.9
-3.86	Short chain dehydrogenase helC	Q4WR19	7	37.7
-3.96	Protein DOM34 homolog	Q4WI62	7	26.6
-3.97	1,3-beta-glucanosyltransferase	Q4WBF7	4	8.9
-4.05	Elongation of fatty acids protein	Q4WEE9	4	16
-4.20	Calcium/calmodulin dependent protein kinase	Q4WXH7	7	25.1
-5.49	Methyltransferase	Q4X081	4	30.3
-6.30	HYPK_UBA domain-containing protein	Q4WPC3	2	17.7
